# Stable Reference Genes for qPCR Analysis in BM-MSCs Undergoing Osteogenic Differentiation within 3D Hyaluronan-Based Hydrogels

**DOI:** 10.3390/ijms21239195

**Published:** 2020-12-02

**Authors:** Johannes Hasler, Luan Phelipe Hatt, Martin James Stoddart, Angela Rita Armiento

**Affiliations:** 1 AO Research Institute Davos, 7270 Davos Platz, Switzerland; jhasler@student.ethz.ch (J.H.); phelipe.hatt@aofoundation.org (L.P.H.); martin.stoddart@aofoundation.org (M.J.S.); 2 Institute for Biomechanics, ETH Zürich, 8093 Zürich, Switzerland

**Keywords:** RT-qPCR, endogenous control, *RPLP0*, *OAZ1*, *PPIA*, biomaterials, osteogenesis, mesenchymal stem cells, tissue engineering, hyaluronic acid

## Abstract

Reverse transcription quantitative polymerase chain reaction (RT-qPCR) enables the monitoring of changes in cell phenotype via the high-throughput screening of numerous genes. RT-qPCR is a fundamental approach in numerous research fields, including biomaterials, yet little attention has been given to the potential impact of 3D versus monolayer (2D) cell culture and to the requirement for a constant validation of the multiple steps of gene expression analysis. The aim of this study is to use high-quality RNA to identify the most suitable reference genes for RT-qPCR analysis during the osteogenic differentiation of human bone marrow mesenchymal stem/stromal cells (BM-MSCs). BM-MSCs are cultured under osteogenic conditions for 28 days in 2D or within hyaluronic acid hydrogels (3D). RNA is subject to quality controls and is then used to identify the most stable reference genes using geNorm, NormFinder, and the ∆Cq method. The effect of the reverse transcriptase is investigated, as well as the expression of osteogenic-related markers. This study shows marked differences in the stability of reference genes between 2D (*RPLP0/GAPDH*) and 3D (*OAZ1/PPIA*) culture, suggesting that it is critical to choose appropriate reference genes for 3D osteogenic cell cultures. Thus, a thorough validation under specific experimental settings is essential to obtain meaningful gene expression results.

## 1. Introduction

Bone marrow-derived mesenchymal stem/stromal cells (BM-MSCs) are certainly among the most studied cell type in the musculoskeletal research field [1]. Their multilineage potential is the continuous object of various investigations, as it suggests the possibility of cell-based therapies to regenerate tissues such as bone and cartilage.

The osteogenic differentiation of BM-MSCs was described for the first time in monolayer culture by Jaiswal and colleagues in the late 1990s [2]. In this study, von Kossa and alkaline phosphatase staining are the main outcome measurements, and the expression of osteogenic marker mRNA is demonstrated by Northern blot. Subsequently, the field of tissue engineering emerged to combine engineering with life science, and BM-MSCs were soon in the spotlight of the tissue engineering triad (cell-material-growth factors) targeting bone regeneration. Monolayer cell cultures are a simple method to gain a first perception of the mechanisms underlying cell differentiation, but they lack the three-dimensional characteristic of tissues. Tissue engineering makes use of natural and synthetic materials to more faithfully recreate the 3D microenvironment and therefore increases the complexity of in vitro culture systems.

A major paradigm shift in life science research occurs with the discovery of the polymerase chain reaction (PCR) [3,4]. The PCR can not only be used to sequence genomic DNA, but is also the most accurate technique to detect changes in gene expression at the mRNA level [5]. Throughout the years, improvements in PCR technology have resulted in the established use of reverse transcription quantitative real-time PCR (RT-qPCR) [6] and labelled/quenched probes such as the TaqMan™ technology to increase detection specificity [7]. Today, gene expression analysis by RT-qPCR is considered the technique par excellence in molecular biology, allowing the high-throughput quantification of a wide range of target genes with a high specificity and sensitivity.

Despite its widespread usage, the successful implementation of RT-qPCR to assess experimental outcomes faces numerous technical challenges that impact assay performance. Sample preparation, RNA quality, RT efficiency, and differences in experimental conditions can introduce significant variations that can wrongly be attributed to a biological effect. To correct for these variables, various papers [8,9,10] highlight the need for the optimization of all these aspects and the necessity of providing more accurate experimental details in publications [8]. A common method with which to analyze qPCR data is the ∆∆Cq method, based on the use of a reference gene as a normalizer. To be used as a normalizer, a reference gene must be stable throughout the duration of the experiment and across groups and should be expressed at a similar level to the target gene(s) of interest. Unfortunately, inappropriate normalization is one of the common pitfalls of qPCR, and often too few details are reported in gene expression studies. The choice of suitable reference genes requires a thorough validation of the different and widely ranging genes involved in various cellular pathways. This is a critical step prior to gene expression analysis, since many reference genes are unstably expressed under varying experimental conditions. However, reference gene stability remains poorly investigated in the presence of drastic differences among experimental groups [9], and the use of just one reference gene is a common but incorrect practice in many studies.

All these concepts have profound implications in biomaterial-assisted cell cultures, where the application of RT-qPCR is increasing as a tool to investigate changes in cell phenotype associated with both cell–cell and cell–material interactions [11]. Three-dimensional cultures fundamentally differ from conventional monolayer, but often the same methods are applied for gene expression studies. For instance, the extraction of high-quality RNA from complex matrixes can be particularly challenging [12,13], especially with increasing culture duration, during which time increasing amounts of extracellular matrix are produced. This is also the case of the hyaluronic acid (HA)-based biomaterials commonly used in tissue engineering studies relevant to the musculoskeletal field [14,15,16]. The three dimensionality of a biomaterial-based culture, together with the composition of HA itself, likely influence the expression of reference gene candidates over a prolonged culture period. We believe that the optimal gene expression analysis strategy for HA-based 3D cultures cannot be simply extrapolated from prior assessments made in conventional monolayer cultures.

This study aims to use high-quality RNA from cells in long-term 3D cultures to identify the most suitable normalizer for gene expression analysis during the osteogenic differentiation of hBM-MSCs within HA-based hydrogels. The identification of suitable reference genes, aside from the traditional ones, highlights the importance of performing a thorough validation under the specific experimental settings to obtain a meaningful gene expression analysis.

## 2. Results

### 2.1. High-Quality RNA Can Be Extracted from BM-MSCs within MeHA Using Double Phase Separation

**Figure 1:**
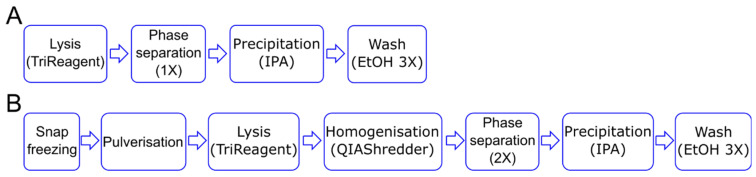
Schematic of RNA isolation steps. (**A**) For the 2D culture, the cell lysis in the TRI Reagent is followed by phase separation and RNA precipitation using isopropanol alcohol (IPA). (**B**) For the 3D culture, the samples are snap frozen and pulverized before the lysis step. The lysate is homogenized and then subject to two rounds of phase separation before proceeding with the RNA precipitation step. In both cases, RNA is washed three times using 70% ethanol (EtOH).

The first step in a gene expression study is the isolation of pure and intact RNA, as both the purity [17] and integrity [18] of RNA affects the performance of downstream applications. Given the 3D nature of the methacrylated HA (MeHA) hydrogels, and the long-term culture period under osteogenic culture conditions (28 days), we modify the standard Tri-Reagent-based protocol to extract RNA from cells in monolayer (Figure 1A) by adding additional steps generally required when working with mammalian tissues. To obtain the complete disruption of the sample, the hydrogels are snap frozen in liquid nitrogen and pulverized prior to lysis. To reduce the viscosity of the lysate, a homogenization step is also added before a double phase separation is performed (Figure 1B).

To calculate the amount of isolated RNA from the 3D cultures and assess its purity, we perform absorbance measurements using a NanoDrop^®^ spectrophotometer. In common laboratory practice, RNA samples with A_260_/A_280_ and A_260_/A_230_ ratios ≥1.8 are considered clean from contaminants absorbing at 280 and 230 nm, with the absorbance at 230 nm known to be negatively affected by low concentrations of RNA. The RNA of all the samples has a A_260_/A_280_ ratio above 1.80. The A_260_/A_230_ ratio is affected by the reduction in total RNA at day 28, with values above 1.75 only for the samples at day 0. Indeed, there is a consistent ~three-fold reduction in the amount of recovered RNA between day 0 and day 28 from all four BM-MSC donors (Table 1).

**Figure 2:**
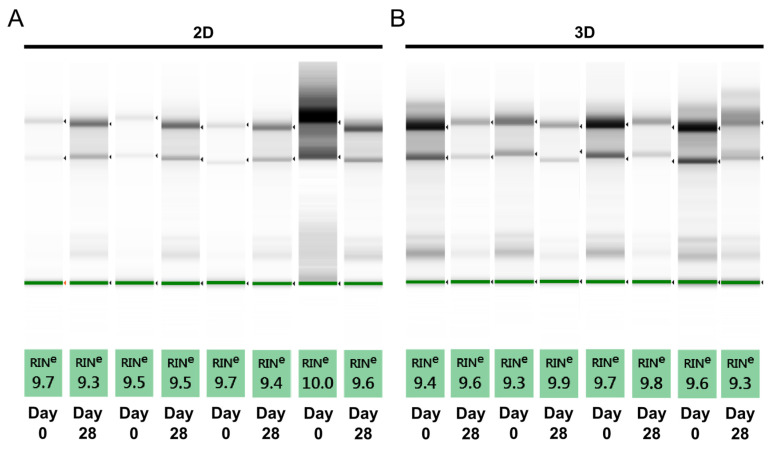
Assessment of RNA quality using RNA integrity number (RIN) equivalent. RNA integrity number is assigned as equivalent RIN using a 2200 TapeStation system. (**A**) One representative sample from each donor in 2D culture; and (**B**) one representative sample from each donor in 3D culture for a total of four donor per each culture type.

Although spectrophotometric assessment provides an indication about possible contamination, a limitation of this method is the inability to distinguish between intact and degraded RNA. Since degraded RNA does not perform well in downstream applications, we perform an additional quality check to determine RNA integrity before proceeding with RT-qPCR. The RNA integrity number (RIN) is assigned using the Agilent TapeStation system, based on a scale from 1 to 10, with 10 being the highest quality. A RIN of above six results in lower Cq values [19], while degraded RNA results in later gene expression [20] and also affects gene stability [21]. All the RNA samples under investigation have a RIN above nine and compare well with the RNA from a 2D culture (Figure 2).

The utilized protocol allows the isolation of high-quality RNA from MeHA hydrogels maintained under osteogenic culture conditions for 28 days. Both the purity and integrity of the RNA are comparable to those of the RNA obtained from 2D cultures serving as a reference standard, and therefore the material can be used in downstream applications such as RT-qPCR.

### 2.2. The Type of Reverse Transcriptase Affects the ∆Cq Values During Real Time PCR

Once high-quality RNA is obtained from both 2D and 3D cultures, complementary DNA (cDNA) is synthesized using a reverse transcriptase chosen from a variety of commercially available reverse transcriptase enzymes. To determine the effect of the reverse transcriptase on the cycle quantification (Cq) value detected during qPCR, we produce cDNA samples using two different reverse transcriptases, MultiScribe™ and SuperScript™ VILO™, and then amplify the *RPLP0* cDNA using the TaqMan™ Universal Master Mix.

**Figure 3:**
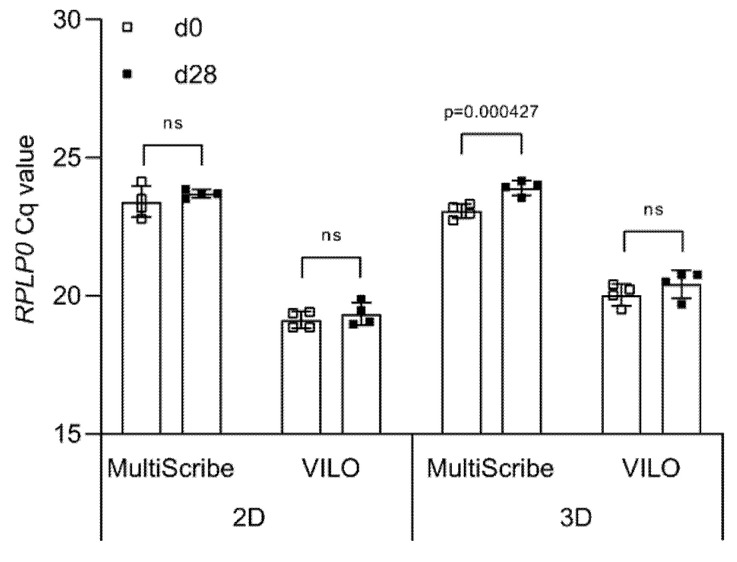
Effect of reverse transcriptase on Cq values. Cq values of *RPLP0* obtained from cDNA synthesized using either MultiScribe or VILO^®^ SuperScript. Data are shown as mean ± SD of independent experiments using cells from four different donors. ns: *p* > 0.05.

In 2D, no statistically significant differences are seen between day 0 (23.39 ± 0.47) and day 28 (23.73 ± 0.33) when using MultiScribe™ (Figure 3). The same is observed for the Cq values obtained when using SuperScript™ VILO™, although the values are lower both at day 0 (19.16 ± 0.39) and at day 28 (19.40 ± 0.40). In 3D, we detect a statistically significant difference between the Cq values at day 0 (23.18 ± 0.31) and day 28 (24.03 ± 0.15) when using MultiScribe™ to obtain cDNA (*p* = 0.000427). The use of SuperScript™ VILO™ reduces the difference in Cq between time points from ΔCq = 0.86 to ΔCq = 0.39, and no statistically significant difference is calculated. As for the 2D, also in 3D the Cq values of *RPLP0* are lower (overall mean 20.23 ± 0.49) when using SuperScript™ VILO™ compared to MultiScribe™ (23.61 ± 0.50).

Overall, we show that the type of reverse transcriptase significantly influences observed differences in the Cq values between time points. In both the 2D and 3D samples, the difference between the Cq values of the two time points is not significantly different when using cDNA synthesized by SuperScript™ VILO™. Therefore, all the upcoming experiments are carried out with cDNA obtained using only SuperScript™ VILO™.

### 2.3. The Culture Type (2D vs. 3D) Influences the Performance of a Reference Gene

A comparative gene expression analysis using the ∆∆Cq method is based on a reference sample and a reference gene to be used as a calibrator and a normalizer, respectively. While the choice of the calibrator is dictated by the experimental groups, the identification of suitable reference genes as a normalizer requires a more careful validation. Changes in culture conditions such as the transition from 2D to 3D and the presence of MeHA can affect the stability of a reference gene. Based on a screening of the literature related to lineage differentiation of hBM-MSCs, we select eight reference genes and amplify them by real-time PCR.

**Figure 4:**
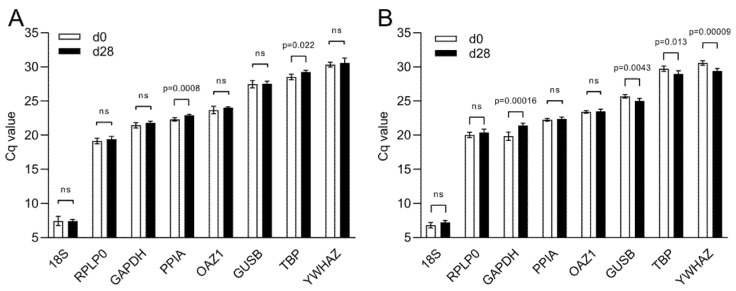
Cq value distribution of eight reference genes during osteogenic differentiation. Cq values of reference genes during osteogenic differentiation of hBM-MSCs in (**A**) 2D culture and in (**B**) 3D culture within MeHA hydrogels. Data are shown as mean ± SD of independent experiments using cells from four different donors. ns: *p* > 0.05.

The Cq values of the candidate reference genes range from a minimum Cq value of 7.43 and 7.03 obtained with *18S* to a maximum of 30.46 and 30.00 for *YWHAZ*, in 2D and 3D culture, respectively (Figure 4). In 2D, *PPIA* and *TBP* are the only two reference genes that show a statistically significant difference between day 0 and day 28 of culture (*p* ≤ 0.0008 and *p* ≤ 0.022, respectively) (Figure 4A). In 3D, *TBP* has different Cq values between the two time points as well as *GAPDH*, *GUSB*, and *YWHAZ*. On the contrary, *PPIA* is stable between time points together with *18S* and *OAZ1* (Figure 4B).

The difference in Cq value between time points can be calculated as ∆Cq (Table 2), while the variation in relation to the mean is expressed by the coefficient of variation (CV).

### 2.4. The Stability of a Reference Gene Differs between 2D and 3D Culture

**Figure 5:**
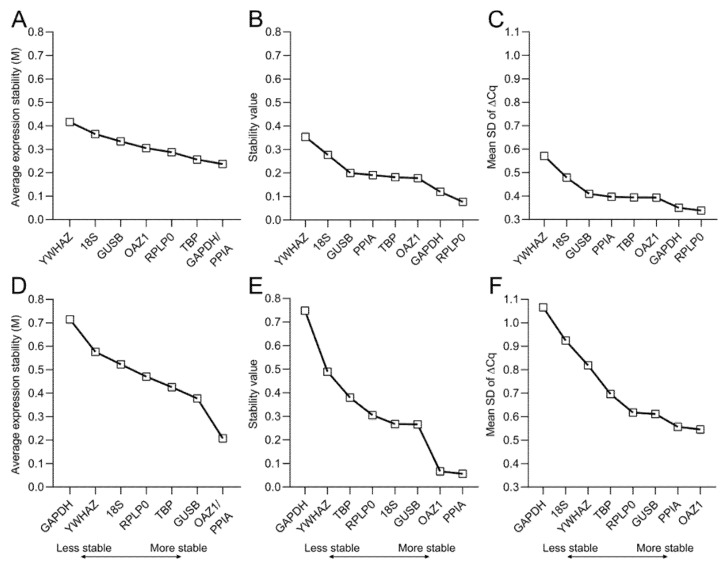
Stability values of the candidate reference genes. Stability of reference genes during the osteogenic differentiation of hBM-MSCs are calculated for both (**A**–**C**) 2D culture; and (**D**–**F**) 3D culture within MeHA hydrogels using (**A**,**D**) geNorm, (**B**,**E**) NormFinder, and (**C**,**F**) the ∆Cq method. The lower the stability value, the higher the average stability of the gene expression over the 28-day culture period.

To effectively evaluate the stability of reference genes, several statistical methods and algorithms have been developed and are freely available. In this work, we make use of three different methods to calculate the average expression stability (geNorm [22]), stability value (NormFinder [23]), and mean SD of ∆Cq (∆Cq method [24]) for both 2D and 3D culture (Figure 5).

**Figure A1:**
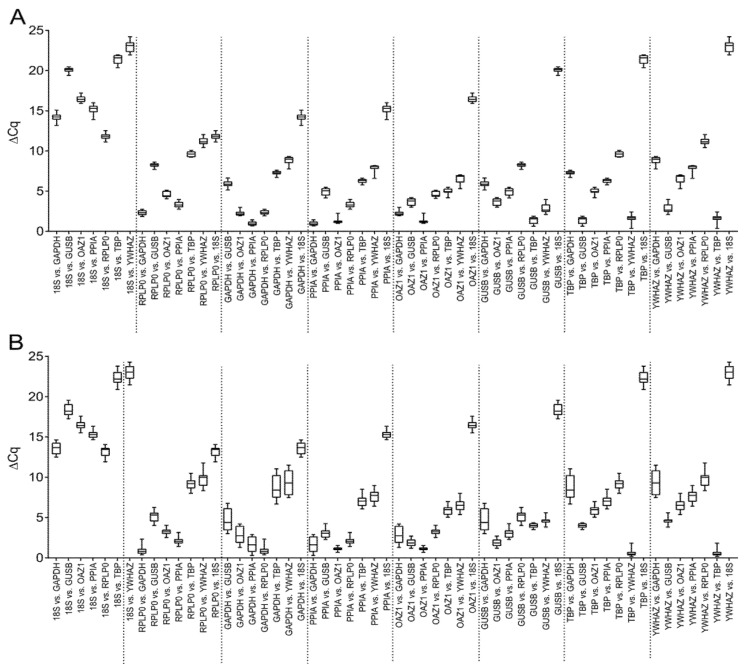
∆Cq approach to reference gene selection. Comparison of reference gene in (**A**) 2D culture; and (**B**) 3D culture. Data are shown as median, 5 to 95 percentiles (boxes) and ranges (whiskers) with independent experiments using cells from four different donors.

According to geNorm, *PPIA* and *GAPDH* are the most stable reference genes for 2D osteogenic cultures of hBM-MSCs, while *YWHAZ* is the least stable gene (Figure 5A). However, in 3D culture *PPIA* and *OAZ1* are the best-performing reference genes. Additionally, in contrast to the 2D data, *GAPDH* has the highest M value, thereby being the least stable reference gene for the osteogenic culture of hBM-MSCs in MeHA (Figure 5D). NormFinder and the ∆Cq method confirm *YWHAZ* as the least stable gene in the 2D data, while they identify *RPLP0* as the best-performing gene together with *GAPDH* (Figure 5B,C and Figure A1). For the 3D samples, NormFinder and the ∆Cq method assign the lowest values to *PPIA* and *OAZ1* and the highest value to *GAPDH* (Figure 5E,F and Figure A1), therefore confirming *PPIA*/*OAZ1* and *GAPDH* as the best- and worst-performing genes, respectively, in osteogenic cultures within MeHA hydrogels.

A ranking of the reference genes is made according to the stability/SD values to ease the comparison across methods and, to gain an overall ranking, the geometric mean of the values obtained with the three different methods is also calculated (Table 3).

**Figure 6:**
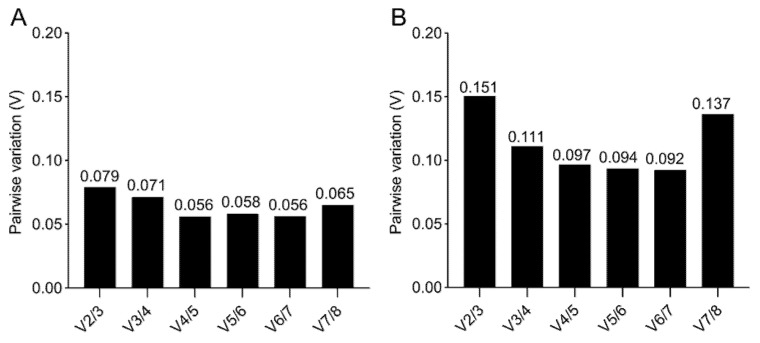
geNorm pairwise comparison. Determination of the minimum number of reference genes required for accurate normalization for 2D (**A**,**B**) 3D culture, obtained by pairwise variation with a cut-off value of 0.15.

A further value (V) is calculated, based on a pairwise comparison in geNorm (Figure 6), to obtain the optimal number of reference genes required for a robust normalization. When the V of the specific pairwise variation is ≥0.15, the addition of a further reference gene has a significant effect. While, for the 2D culture, a minimum of two reference genes is sufficient for a reliable gene expression analysis (Figure 6A), for the 3D culture the pairwise comparison suggests that three reference genes are required, since the V2/3 comparison has a V= 0.151 (Figure 6B).

The validation of the eight reference gene candidates highlights the larger spread in stability across the reference genes in samples from 3D cultures compared to the one from 2D cultures. The results obtained using three different methods are comparable. They identify *RPLP0* and *GAPDH* as the most suitable reference genes for the 2D osteogenic culture of BM-MSCs, and *PPIA*, *OAZ1*, and *GUSB* as the top three reference genes for the gene expression analysis of osteogenically differentiated BM-MSCs within MeHA hydrogels.

### 2.5. The Reference Gene of Choice Has an Impact on the Target Gene Expression

To stress the importance of choosing the appropriate reference gene, the expression of two osteogenic target genes is calculated using the samples at day 0 as a calibrator and the different reference genes under validation as a normalizer.

**Figure 7:**
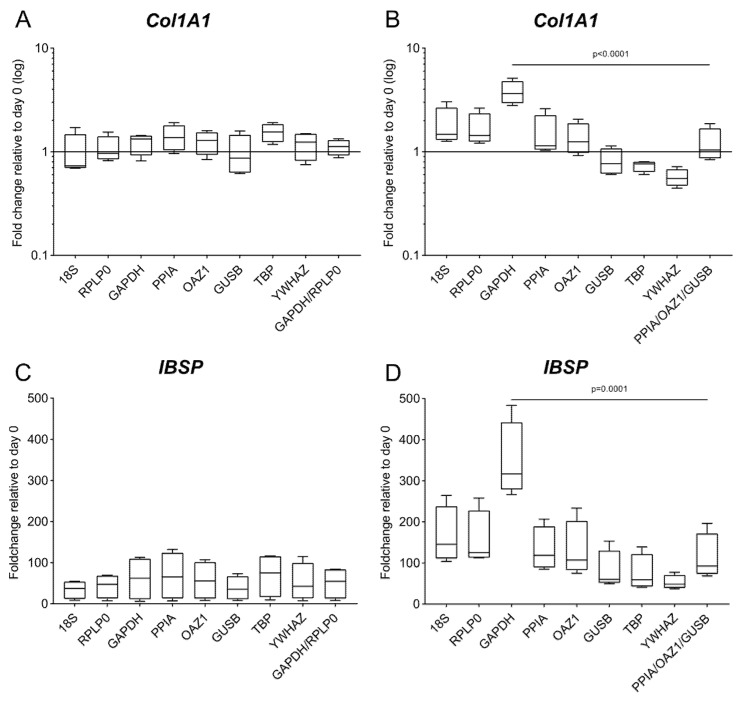
Gene expression of *IBSP* and *Col1A1* using different reference genes. Expression of *Col1A1* and *IBSP* was analyzed in (**A**,**C**) 2D culture and (**B**,**D**) 3D culture at day 28 of osteogenic differentiation. Analysis is performed according to the 2^−∆∆Cq^ method using each of the eight candidate genes as a reference gene and the day 0 reference sample. The geometric mean of the top 2 and 3 genes according to the geNorm algorithm is used as a comparison for the 2D and the 3D culture, respectively. Data are shown as mean ± SD of independent experiments using cells from four different donors.

For both culture types, the expression of both *Col1A1* and *IBSP* is affected by the chosen reference gene (Figure 7), in particular for the 3D culture, where the fold change of *Col1A1* has opposite trends and *IBSP* has a fold change varying between 52.9 and 345.87 depending on the choice of reference gene. Based on the results of the comprehensive ranking, we use the geometric mean of the two (*GAPDH*/*RPLP0*, for 2D) or three (*PPIA*/*OAZ1*/*GUSB*, for 3D) reference genes with the lowest mean of all stability values for the analysis of the target genes [22]. When compared to data obtained using the worst-performing reference gene (*YWHAZ* in 2D and *GAPDH* in 3D), a statistically significant difference is detected only for the 3D culture (*p* < 0.0001 for *Col1A1* and *p* = 0.0001 for *IBSP*) (Figure 7B,D).

The differential impact that the choice of reference genes has on the target gene expression in 2D and in 3D confirms the results of the validation, where the range of stability is higher in the 3D culture compared to the 2D setting (Figure 5).

### 2.6. Osteogenic Differentiation of hBM-MSCs within MeHA Hydrogels

To conclude our study, we use the identified reference genes to perform a gene expression analysis on a selected number of target genes commonly used to assess the osteogenic differentiation of hBM-MSCs.

**Figure 8:**
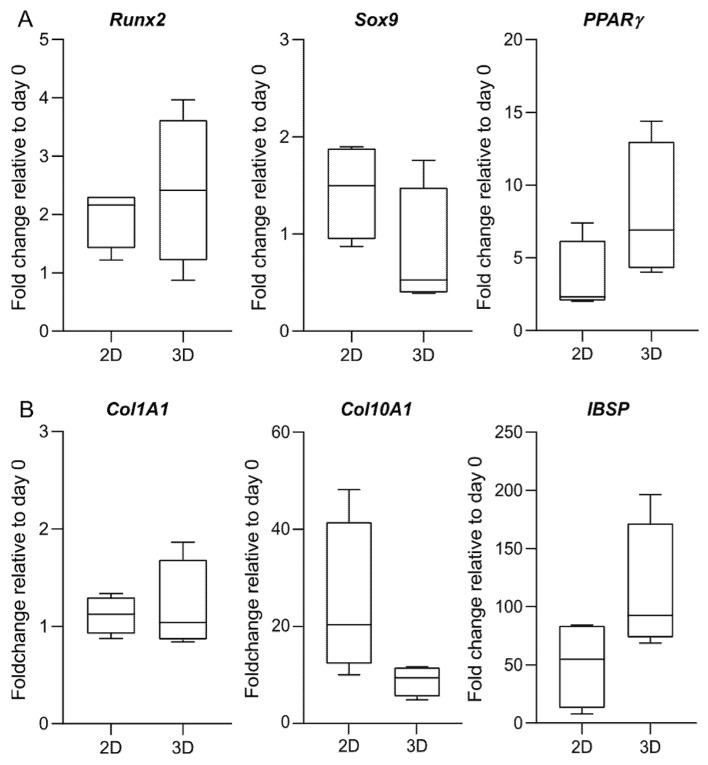
Gene expression of osteogenic-related genes. Expression of (**A**) transcription factors; and (**B**) osteogenic markers is analysed in 2D and 3D culture at day 28 of osteogenic differentiation. Analysis is performed according to the 2^−∆∆Cq^ method, with day 0 used as a reference sample and the geometric mean of *RPLP0* and *GAPDH* or *OAZ1*, *PPIA*, and *GUSB* used as an endogenous control for the 2D and the 3D data, respectively. Data are shown as the median, 5 to 95 percentiles (boxes), and ranges (whiskers) of independent experiments using cells from four different donors. There is no statistically significant difference in the fold change between the 2D and the 3D culture.

Consistent with cells in a monolayer, we observe an increased expression of the late-stage osteogenic marker *IBSP* at day 28, confirming the osteogenic commitment of the cells in 3D cultures of MeHA hydrogels (Figure 8). The expression at the mRNA level of all the genes under investigation is comparable between the 2D and the 3D culture. No statistical significance can be identified between the 2D and the 3D culture in the fold changes of the target genes. However, when compared to osteogenic 2D culture, there is a clear trend for cells within MeHA to express lower levels of *Sox9* and *Co10A1* and increased levels of *PPARγ* and *IBSP*.

## 3. Discussion

Tissue engineering and RT-qPCR have both revolutionized life science research by facilitating the transition from 2D to 3D culture systems and allowing the high-throughput screening of mRNA expression, respectively. In particular, the tantalizing regenerative properties of BM-MSCs for cell-based musculoskeletal regenerative approaches has seen intensive research interest in recent years. However, despite the large number of promising in vitro and preclinical approaches, MSC-based therapies have yet to make a significant impact in the clinical setting. This phenomenon is partly due to difficulties in translating methodologies and experimental findings, including gene expression results, from conventional 2D cultures to 3D biomaterial- and organ-based cultures.

With this in mind, we have utilized 2D and 3D osteogenic cultures of BM-MSCs to validate all the steps of a typical gene expression study, from RNA extraction to the analysis of osteogenic-related markers. We have demonstrated that the 3D osteogenic culture of human BM-MSCs within MeHA hydrogels requires an improved RNA extraction method and the use of different sets of reference genes compared to conventional monolayer/2D osteogenic cultures. The results of this study provide a direct comparison between 2D and 3D culture conditions that highlights the importance of appropriate validation steps to obtain biologically meaningful data representative of the cell type, the culture conditions used, and the type of biomaterial under investigation.

Accurate gene expression analysis can only be performed following the validation of a broad range of reference genes involved in different aspects of cellular activities to minimize the effect of variation induced by the experimental conditions [10]. In our specific experimental context, we use one cell type and ensure consistency in osteogenic differentiation conditions and experimental duration to specifically assess the impact of 2D vs. 3D culture conditions on reference gene expression. Previous work from Cagnan and colleagues [25] identifies *RPLP0* and *GAPDH* as suitable reference genes during the differentiation of bone marrow-derived MSCs (both osteogenic and adipogenic). The stability of *RPLP0* has also been confirmed in various studies of monolayer cultures across different MSC tissue sources and differentiation potentials [26,27,28], which is confirmed in our experimental settings. In a study screening various tissue sources, *GAPDH* expression stability displays high variability in expression between different types of tissue but expression levels within related tissues are comparable [29]. Consistent with this, our study demonstrates that *GAPDH* is a stable reference gene, but in a context-dependent manner. Indeed, while *GAPDH* stability is high in 2D, this is not the case in 3D HA-based cultures. This demonstrates that the sensitivity of *GAPDH* is influenced by the 3D environment, as already seen in human BM-MSCs cultured within peracetic acid-treated human cancellous bone cubes [30]. In addition, the observations of Quiroz et al. have shown that *GAPDH* expression is also subject to osteogenic regulation during monolayer differentiation [31]. In our study, no such upregulation is evident in monolayer cultures, although the high ΔCq value observed for *GAPDH* confirms its upregulation during osteogenic differentiation at day 28 for the 3D culture.

In 3D HA-based studies, the geometric mean of *OAZ1, PPIA*, and *GUSB* serves as the most suitable combination of reference genes for use as a normalizer. *PPIA* has previously been reported as a stable reference gene during the osteogenic differentiation of human BM-MSCs both in 2D [26] and in 3D cultures in cancellous bone explants [30]. Interestingly, in a 3D chondrogenic culture of synovium-derived porcine MSCs, *PPIA* is ranked as highly stable within alginate but very unstable in agarose [32]. This comparison suggests that stability is not only influenced by the three dimensionality of the material but also by its biophysical properties, since alginate is negatively charged while agarose has a neutral charge. This finding could be transferred to our results, where *PPIA* behaves as highly stable in a negatively charged polysaccharide culture. A screening of more than 13,000 genes from diverse tissues and experimental conditions has established *OAZ1* as a top reference gene [33], thus supporting our findings of *OAZ1* as being a suitable endogenous control for 3D cultures. *OAZ1* has been identified as a suitable reference gene in studies involving a high protein turnover [34] and during the development of thymic epithelial cells [35], but the literature on its efficacy as a reference gene in MSCs is scarce. *GUSB* has also been shown to be among the best performing reference genes for human MSC tri-lineage differentiation [26]. Utilizing the same validation methods as in our study, Brinkdorf et al. has ranked *GUSB* as one of the least stable genes for immortalized human MSCs in 2D and 3D. Conversely, in the same study, BestKeeper identifies *GUSB* as the most stable reference gene, demonstrating that stability can differ among methods and an overall ranking is required [36]. Various studies have also suggested that *YWHAZ* may be a suitable reference gene in monolayer cultures [26,37], however we observe that *YWHAZ* is not an appropriate choice of reference gene in 2D and 3D HA-based osteogenic cultures, which supports the findings of Brinkdorf et al. [36] that *YWHAZ* is not suited for 3D culture. A further point concerns the commonly used reference gene *18S*, which we consider as an unsuitable choice as a reference gene, despite its apparent stability in 3D cultures. The extremely high expression of *18S*, very far from the Cq of most of the genes of interest, and its ribosomal nature (and thus its absence in highly purified mRNA samples), indicates that *18S* should not be considered as an appropriate choice of reference gene in future studies [9,31].

One could argue that the choice of 3D culture system used in this study does not specifically permit the determination of the contribution of the 3D setting per se from the intrinsic properties of HA for influencing the stability of reference gene expression. To further investigate this, cells could be seeded onto the surface of the MeHA hydrogel to determine how HA-mediated signaling influences reference gene expression. However, the known anti-adhesive properties of HA-based hydrogels [38] would require a further modification of the biomaterial to allow reproducible seeding on its surface. As such, the 2D monolayer culture was chosen for our study, given its established use in BM-MSC studies.

Despite the anti-adhesive nature of HA-based hydrogels, MSC laden HA-based hydrogels are promising approaches for tissue engineering strategies. HA is synthesized at the site of the future joint [39] and it is present within the cartilaginous callus during endochondral fracture repair [40]. In support of the osteogenic properties of our HA-based material for bone tissue engineering, a key determinant of early fate decision of MSCs is the transcription factor *Sox9*, which acts as an inhibitor of *Runx2* expression during chondrogenesis [41,42]. The downregulation of *Sox9* is therefore required for the onset of osteogenesis, and its expression trend in BM-MSCs observed within our MeHA hydrogel confirms the osteogenic phenotype of the cells. In addition, we observed a consistently increased expression of the late-stage osteogenic marker *IBSP* at day 28 in 3D cultures of our MeHA hydrogels. This is consistent with the findings of Zou and colleagues, who demonstrated that HA possesses good intrinsic osteogenic properties, with a predominant positive role in early and late markers of bone formation [43]. Previous work from Rauh et al. [30] also reported that *IBSP* is upregulated in 3D cultures of peracetic acid-treated human cancellous bone explants after 14 days. The marked upregulation of *IBSP* mRNA in our study would suggest that this is an effect induced by the 3D culture environment rather than the HA itself. The expression of *Col10A1*, an extracellular matrix protein expressed by hypertrophic chondrocytes [44], also appears to be affected by the 3D setting. In a recent study, where HA is supplemented to the chondrogenic culture medium of human BM-MSCs in fibrin/polyurethane scaffolds, the expression of *Col10A1* is reduced compared to untreated scaffolds [45]. In the light of these findings, the expression of *Col10A1* could be driven by the presence of HA rather than the 3D culture itself.

It could be considered that certain limitations are evident in this study, such as the use of only one cell type and the investigation of only one HA-based biomaterial under osteogenic culture conditions. However, given the widespread use of MSCs and HA-based hydrogels in tissue reparative approaches [46,47] and the known inter-individual heterogeneity of MSCs, the robust nature of our findings suggest that the use of the identified reference genes may serve to improve RT-qPCR-based assessments of phenotypic changes in osteogenic cultures. Further studies to determine the most appropriate reference genes for other MSC-relevant differentiation protocols (such as chondrogenesis), and when utilizing other widely used hydrogels (such as fibrin or collagen- and gelatin-based materials), should also be performed as a matter of urgency in an attempt to advance the field of MSC-based musculoskeletal regenerative approaches.

In conclusion, we have identified a robust set of reference genes for both 2D and 3D investigations concerning the osteogenic differentiation of BM-MSCs in HA-based materials. In addition, our study also provides a method to obtain high-quality RNA from challenging HA-based materials and stresses the importance of a thorough validation of reference genes to be conducted prior to the assessment of gene expression changes in 3D biomaterial cultures.

## 4. Materials and Methods

The bone marrow aspirates used in this study were obtained upon the informed consent of the donors with full approval from the Ethics Committee of the University of Freiburg Medical Centre (EK-Freiburg: 135/14) and the ethical commission of Graubünden (KEK-ZH-NR: 2016-00141).

All the reagents were purchased from Sigma-Aldrich (St. Luis, MO, USA) unless otherwise stated.

### 4.1. Methacrylated Hyaluronic Acid Polymer Synthesis

MeHA was synthesized according to [48,49] and stored at −20 °C until use. The degree of substitution was measured by nuclear magnetic resonance.

### 4.2. Cell Isolation and Culture

Human BM-MSCs were isolated and cryopreserved according to an established protocol [50]. Upon thawing, the cells were expanded as described in Hatt et al. [51] Briefly, BM-MSCs were seeded at 3 × 10^3^ cells/ cm^2^ in T300 tissue culture flasks (TPP, Trasadingen, Switzerland) in alpha minimal essential medium eagle (αMEM, Gibco, Carlsbad, CA, USA) supplemented with 10% (*v*/*v*) MSC-qualified fetal bovine serum (FBS; SeraPlus, PAN-Biotech, Aidenbach, Germany), 100 U/mL and 100 µg/mL penicillin and streptomycin (Gibco), respectively, and 5 ng/mL basic fibroblast growth factor (Fitzgerald Industries International, North Acton, MA, USA). Cells were used up to passage 4. Donor details of the hBM-MSCs used in this study are as follows: Donor 1–55-year old female; Donor 2–69-year old female; Donor 3–80-year old male; Donor 4–22-year old female, all from spine vertebral body aspirates.

### 4.3. Cell Laden Methacrylated Hyaluronic Acid Hydrogel Preparation

Irgacure 0.3% (*w*/*v*) in phosphate buffered saline (PBS) was sterile filtered and used as a photoinitiator for the MeHA polymers (47% degree of functionalisation). MeHA, 2% (*w*/*v*), was dissolved in Irgacure solution and kept at 37 °C until use. Under sterile conditions, hBM-MSCs were added to the MeHA hydrogel precursor at a final density of 20 × 10^6^ cells/mL. Cylindrical cell-laden MeHA hydrogels (100 µL volume; containing 2 × 10^6^ cells) were produced in 4% (*w*/*v*) agarose molds (Lonza, Basel, Switzerland), created using a 6 mm diameter biopsy punch (Kai Europe GmbH, Solingen, Germany. MeHA hydrogels were subsequently crosslinked in a BioLink^®^ BLX 365 irradiation system (Witec AG, Sursee, Switzerland) for 1.5 min at 1 J. To reduce cell attachment to plastic during culture, the crosslinked hydrogels were transferred to a silicone mold within a 12-well plate and covered by 1 mL of culture medium.

### 4.4. Osteogenic Differentiation of 2D and 3D Culture

The osteogenic differentiation of the monolayer culture was performed as previously described in Hatt et al. [51]. Briefly, BM-MSCs were cultured in osteocontrol (OC) medium for the first 24 h (Dulbecco’s Modified Eagle Medium (DMEM 1 g/L glucose, Gibco), 10% FBS (Gibco), and 100 U/mL plus 100 µg/mL penicillin and streptomycin, respectively). The OC medium was then supplemented with an osteogenic (OC) cocktail including 10 nM dexamethasone, 50 µg/mL ascorbic acid 2-phosphate, and 10 mM β-glycerophosphate. The first media change is defined as day 0 and the osteogenic culture was maintained for 28 days, with a media change three times per week. All the cultures were performed with four independent donors, with two replicates for each experimental group. Cells were incubated under standard cell culture conditions (37 °C and 5% CO_2_ in a humidified atmosphere).

The cell-laden hydrogels are pre-incubated in OC medium for an initial 24 h period. After this period (defined as experimental day 0), the OC medium was supplemented for a further 28 days with the OG cocktail. As described for the monolayer culture, medium change was performed three times per week, and cultures were repeated with four independent donors and two replicates for each experimental group.

### 4.5. RNA Isolation from 2D and 3D Culture

Cells in monolayer culture were harvested at day 0 and 28 and RNA is isolated according to the TriReagent-based procedure described in Hatt et al. [51].

Cell-laden hydrogels were harvested at days 0 and 28. Each hydrogel was quickly rinsed in PBS, transferred to an Eppendorf tube (Eppendorf, Hamburg, Germany), snap frozen in liquid nitrogen, and stored at −80°C until use. On the day of the RNA extraction, each hydrogel was pulverized using a custom-made pestle and mortar, with further pulverization using a pellet pestle to grind the gel while keeping the samples frozen. The pulverized samples were lysed in TRI-Reagent^®^ (1 mL/sample, supplemented with 5 µL/mL of Polyacryl Carrier; both Molecular Research Center Inc., Cincinnati, OH, USA) with an incubation of 10 min at 4 °C. Sample lysate was then added to QIAShredder columns (Qiagen, Venlo, The Netherlands) and centrifuged at 20,000 g for 5 min. For the phase separation, the homogenate was supplemented with 100 µL 1-bromo-3-chloropropane (BCP), mixed for 15 s, incubated for 15 min at room temperature, then finally centrifuged at 12,000 g for 15 min at 4 °C. Following centrifugation, 80% of the aqueous phase was transferred into a fresh Eppendorf tube. All the steps of the phase separation were repeated a second time and the new aqueous phase was transferred into a fresh Eppendorf tube. An equal volume of isopropanol was then added to precipitate the RNA and the samples were placed on an orbital shaker for 10 min before centrifugation at 12,000 g for further 10 min at 4°C. The RNA pellet was washed three times with 75% ethanol. The ethanol is removed, and the pellet left to air dry for approximately 5 min. The RNA was then dissolved in 20 µL of DEPC-treated water before quantitative and qualitative measurements, then stored at −80 °C until use.

### 4.6. RNA Assessment

To quantify the amount and purity of the recovered RNA, absorbance measurements were carried out with a NanoDrop^TM^ 1000 spectrophotometer (Thermo Scientific, Waltham, MA, USA). The obtained A_260_/A_280_ and A_260_/A_230_ ratios were used to assess the purity of the RNA samples.

The RNA integrity was calculated as the RIN equivalent (RIN^e^) using an RNA ScreenTape kit (Agilent Technologies, Inc, Santa Clara, CA, USA) according to the manufacturer’s instructions. Measurements were performed and visualized using a 2200 TapeStation system (Agilent Technologies) equipped with the Agilent Software Package.

### 4.7. qRT-PCR

Gene expression analysis was performed using two-step qRT-PCR. Total RNA (250 ng in 20 µL, or 500 ng in 40 µL) was retrotranscribed using random hexamers and a MultiScribe^TM^ Reverse Transcriptase (Applied Biosystems, Waltham, MA, USA) or SuperScript™ VILO™ (Invitrogen, Waltham, MA, USA), according to the manufacturer’s instructions. cDNA was diluted 1 to 5 using 1X Tris-EDTA buffer and stored at −20 °C until further use. For simplicity, the products of the two cDNA syntheses are named after the reverse transcriptase used: MultiScribe and VILO.

Real time qPCR was performed using the TaqMan™ Universal Master Mix with 5 ng of cDNA in a 10 µL reaction volume. To exclude contamination and primer-dimer formation, a non-template control was used for each gene under investigation. Each PCR reaction was run in technical triplicates for 40 cycles using a QuantStudio^TM^ 6 Pro Real-Time PCR System (Applied Biosystems^TM^, Foster City, CA, USA). Primers used for the reference genes are listed in Table 4, and the primers of osteogenic target genes are listed in Table 5. A standard deviation (SD) of ≤0.25 is set as threshold for the technical triplicates. Data analysis of osteogenic-related gene expression was performed according to the ΔΔCt method using the geometric mean of *RPLP0* and *GAPDH* for the monolayer culture, and *OAZ1, PPIA,* and *GUSB* for the 3D cell-laden hydrogel culture, as a normalizer and day 0 samples as a calibrator.

### 4.8. Reference Gene Validation

Reference genes (Table 4) were selected based on previous literature [26,33,52] and *18S* and *GAPDH* were added as frequently used reference genes. To minimize the possible regulation of the reference gene during in vitro osteogenic differentiation and/or possible co-regulation within the reference gene, the selected reference genes cover a variety of cellular activities.

### 4.8.1. geNorm

The Cq values of candidate reference genes are transformed by applying the $2^{-\Delta Cq}$ method, where the lowest Cq value is used as a reference. Obtained values are loaded into geNorm v. 3.5 Visual Basic Application for Microsoft Excel. A stability value (M) was computed for all candidate reference genes through a pairwise comparison. Step by step, the highest stability value was excluded, and the average expression stability was recalculated, thereby enabling the ranking of reference genes from the lowest stability value to the most stable combination of reference genes. Furthermore, geNorm suggests the optimal number of reference genes based on the calculation of a new normalization factor ($NF_{n+1}$) on each occasion when the next stable reference gene is added. Between the sequential normalization factors, according to the descending stability values the pairwise variation $V_{n/n+1}$ was then calculated. If the pairwise variation is ≥0.15 when including the (*n* + 1) gene, the addition of that reference gene has a significant effect [22].

#### 4.8.2. NormFinder

NormFinder v0.953 is an Excel based Add-In and, in contrast to geNorm, is a model-based approach. The inclusion of at least eight samples per group and 5–10 candidate reference genes is recommended. Cq values are first calculated as described for geNorm. For the analysis, no grouping is applied and NormFinder is then used to calculate the stability values for all reference genes, with the ranking calculated according to the lowest value [23].

#### 4.8.3. ΔCq Method

Another approach to identify the most stable reference gene is developed by Silver et al. [24]. With this method, the Cq value of pairs of reference genes within a sample is compared by first calculating the mean ΔCq value of each possible combination of pairs of genes and then the SD of the ΔCq (Table A1). The candidate reference genes are then ranked according to the calculated mean SD.

### 4.9. Statistics

Statistical significance between two groups was examined by applying student’s t-test using the Holm–Sidak method, with *p ≤* 0.05 considered statistically significant. For more than two different groups, the statistical significance was examined by applying a one-way ANOVA using Sidak’s multiple comparison test. Statistical analysis was performed using GraphPad Prism 8.1.0 (GraphPad Software Inc., San Diego, CA, USA).

## Figures and Tables

**Table 1:** RNA absorbance measurements using a NanoDrop^®^ spectrophotometer.

Donor	Time Point (Day)	Concentration (ng/µL)	A_260/280_	A_260/230_	Total RNA (ng)
1	0	888.20	1.99	1.79	17,764
	28	278.85	1.97	1.09	5577
2	0	825.61	1.99	1.75	16,512
	28	231.58	1.97	0.91	4632
3	0	769.96	1.99	1.76	15,399
	28	269.77	1.97	1.25	5395
4	0	626.97	1.86	1.80	12,539
	28	222.26	1.80	1.16	4445

**Table 2:** Cq value, ∆Cq, and CV of the candidate reference genes.

	2D				3D			
Gene Symbol	Cq Day 0 ^1^	Cq Day 28 ^1^	∆Cq	CV ^2^	Cq Day 0 ^1^	Cq Day 28 ^1^	∆Cq	CV ^2^
*18S*	7.43 ± 0.67	7.42 ± 0.24	−0.02	6.71	6.81 ± 0.40	7.25 ± 0.25	0.44	5.66
*RPLP0*	19.16 ± 0.39	19.40 ± 0.40	0.24	2.07	20.04 ± 0.40	20.39 ± 0.51	0.35	2.36
*GAPDH*	21.45 ± 0.39	21.81 ± 0.22	0.36	1.65	19.85 ± 0.62	21.41 ± 0.36	1.56	4.57
*PPIA*	22.32 ± 0.23	22.92 ± 0.15	0.60	1.60	22.26 ± 0.20	22.39± 0.28	0.13	1.09
*OAZ1*	23.69 ± 0.54	24.05 ± 0.10	0.36	1.76	23.42 ± 0.20	23.48 ± 0.34	0.06	1.15
*GUSB*	27.48 ± 0.54	27.54 ± 0.36	0.06	1.64	25.69 ± 0.26	25.03 ± 0.35	−0.66	1.78
*TBP*	28.53 ± 0.40	29.26 ± 0.26	0.72	1.73	29.74 ± 0.38	28.96 ± 0.46	−0.78	1.96
*YWHAZ*	30.34 ± 0.35	30.58 ± 0.72	0.25	1.83	30.58 ± 0.34	29.42 ± 0.36	−1.16	2.29

^1^ Data are shown as mean± SD; ^2^ data are shown as %.

**Table 3:** Ranking of all reference genes according to the different methods.

**2D**				
**Gene Symbol**	**geNorm**	**NormFinder**	**ΔCq Method**	**Comprehensive Ranking ^1^**
*RPLP0*	3	1	1	1
*GAPDH*	1	2	2	2
*TBP*	2	4	3	4
*GUSB*	5	6	6	6
*OAZ1*	4	3	4	5
*PPIA*	1	5	5	3
*18S*	6	7	7	7
*YWHAZ*	7	8	8	8
**3D**				
**Gene Symbol**	**geNorm**	**NormFinder**	**ΔCq Method**	**Comprehensive Ranking ^1^**
*OAZ1*	1	2	1	2
*PPIA*	1	1	2	1
*GUSB*	2	3	3	3
*TBP*	3	6	5	5
*RPLP0*	4	5	4	4
*18S*	5	4	7	6
*YWHAZ*	6	7	6	7
*GAPDH*	7	8	8	8

^1^ Data are shown as the geometric mean of the three stability values.

**Table 4:** Candidate reference genes under investigation.

Gene Symbol	Assay ID ^1^	Forward/Reverse/Probe
*18S*	Hs99999901_s1	
*GAPDH*	Hs99999905_m1	
*GUSB*	Hs99999908_m1	
*OAZ1*	Hs00427923_m1	
*PPIA*	Hs99999904_m1	
*RPLP0*		5′-TGG GCA AGA ACA CCA TGA TG-3′ 5′-CGG ATA TGA GGC AGC AGT TTC-3′ 5′-AGG GCA CCT GGA AAA CAA CCC AGC-3′
*TBP*	Hs00427620_m1	
*YWHAZ*	Hs00237047_m1	

*18S*: *18S* ribosomal RNA, *GAPDH*: Glyceraldehyde-3-phosphate dehydrogenase, *GUSB*: Beta-glucuronidase, *OAZ1*: Ornithine Decarboxylase Antienzyme 1, *PPIA*: Peptidylpropyl Isomerase A, *RPLP0*: Ribosomal Protein Lateral Stalk Subunit P0, *TBP*: TATA box binding protein, *YWHAZ*: Tyrosine 3-Monooxygenase/Tryptophan 5-Monooxygenase Activation Protein Zeta. ^1^ TaqMan^®^ Gene Expression Assay (Applied Biosystems).

**Table 5:** Target genes under investigation.

Gene Symbol	Assay ID ^1^	Forward/Reverse/Probe
*ALPL*	Hs00758162_m1	
*COL1A1*		5′-CCC TGG AAA GAA TGG AGA TGA T-3′ 5′-ACT GAA ACC TCT GTG TCC CTT CA-3′ 5′-CGG GCA ATC CTC GAG CAC CC-3′
*COL10A1*		5′-ACG CTG AAC GAT ACC AAA TG-3′ 5′-TGC TAT ACC TTT ACT CTT TAT GGT GTA-3′ 5′-ACT ACC CAA CAC CAA GAC ACA GTT CTT CAT TCC-3′
*IBSP*	Hs0017320_m1	
*PPARγ*	Hs00234592_m1	
*RUNX2*		5′-AGC AAG GTT CAA CGA TCT GAG AT-3′ 5′-TTT GTG AAG ACG GTT ATG GTC AA-3′ 5′-AGG GCA CCT GGA AAA CAA CCC AGC-3′
*SOX9*	Hs00165814_m1	

*ALPL*: Alkaline phosphatase, *COL1A1*: Collagen type 1, *COL10A1*: Collagen type 10, *IBSP*: Bone sialoprotein, *PPARγ:* Peroxisome proliferator-activated receptor gamma, *RUNX2*: Runt-related transcription factor 2, *SOX9*: Sex determining region Y box 9. ^1^ TaqMan^®^ Gene Expression Assay (Applied Biosystems).

## Abbreviations

*ALPL*	Alkaline phosphatase
BCP	1-Bromo-3-chloropropane
BM-MSCs	Bone marrow-derived mesenchymal stromal cells
*COL1A1*	Collagen type 1
*COL10A1*	Collagen type 10
Cq	Cycle quantification
∆Cq	Delta cycle quantification
EtOH	Ethyl alcohol
FBS	Fœtal bovine serum
*GAPDH*	Glyceraldehyde-3-phosphate dehydrogenase
*GUSB*	Beta-glucuronidase
HA	Hyaluronic acid
*IBSP*	Bone sialoprotein
IPA	Isopropanol alcohol
MeHA	Methacrylated hyaluronic acid
*OAZ1*	Ornithine Decarboxylase Antizyme 1
OC	Osteocontrol
OG	Osteogenic
PCR	Polymerase chain reaction
PBS	Phosphate buffered saline
*PPARγ*	Peroxisome proliferator-activated receptor gamma
*PPIA*	Peptidylprolyl Isomerase A
*RUNX2*	Runt-related transcription factor 2
RT-qPCR	Reverse transcription quantitative polymerase chain reaction
RIN	RNA integrity number
*RPLP0*	Ribosomal Protein Lateral Stalk Subunit P0
SD	Standard deviation
*SOX9*	Sex determining region Y box 9
*TBP*	TATA box binding protein
*YWHAZ*	Tyrosine 3-Monooxygenase/Tryptophan 5-Monooxygenase Activation Protein Zeta
*18S*	18S ribosomal RNA

## Appendix A

**Table A1:** Gene comparison according to the ∆Cq method.

	2D			3D		
Comparison	∆Cq Mean	SD	Mean SD	∆Cq Mean	SD	Mean SD
*18S* vs. *GAPDH*	14.19	0.49		13.61	0.71	
*18S* vs. *GUSB*	20.08	0.30		18.33	0.70	
*18S* vs. *OAZ1*	16.43	0.39		16.42	0.53	
*18S* vs. *PPIA*	15.16	0.56		15.30	0.43	
*18S* vs. *RPLP0*	11.83	0.38		13.19	0.63	
*18S* vs. *TBP*	21.44	0.53		22.33	0.85	
*18S* vs. *YWHAZ*	22.99	0.70	0.48	22.97	0.89	0.92
*RPLP0* vs. *GAPDH*	2.36	0.26		0.92	0.56	
*RPLP0* vs. *GUSB*	8.25	0.24		5.15	0.66	
*RPLP0* vs. *OAZ1*	4.61	0.34		3.24	0.36	
*RPLP0* vs. *PPIA*	3.33	0.37		2.11	0.44	
*RPLP0* vs. *TBP*	9.61	0.33		9.14	0.69	
*RPLP0* vs. *YWHAZ*	11.16	0.45		9.79	0.98	
*RPLP0* vs. *18S*	11.83	0.38	0.34	13.19	0.63	0.62
*GAPDH* vs. *GUSB*	5.89	0.38		4.73	1.33	
*GAPDH* vs. *OAZ1*	2.25	0.29		2.82	1.03	
*GAPDH* vs. *PPIA*	0.97	0.24		1.69	0.92	
*GAPDH* vs. *RPLP0*	2.36	0.26		0.92	0.56	
*GAPDH* vs. *TBP*	7.25	0.27		8.72	1.44	
*GAPDH* vs. *YWHAZ*	8.80	0.52		9.37	1.48	
*GAPDH* vs. *18S*	14.19	0.49	0.35	13.61	0.71	1.76
*PPIA* vs. *GAPDH*	0.97	0.24		1.69	0.92	
*PPIA* vs. *GUSB*	4.92	0.47		3.03	0.50	
*PPIA* vs. *OAZ1*	1.27	0.32		1.12	0.21	
*PPIA* vs. *RPLP0*	3.33	0.37		2.11	0.44	
*PPIA* vs. *TBP*	6.28	0.26		7.03	0.64	
*PPIA* vs. *YWHAZ*	7.83	0.55		7.68	0.75	
*PPIA* vs. *18S*	15.16	0.56	0.40	15.30	0.43	0.56
*OAZ1* vs. *GAPDH*	2.25	0.29		2.82	1.03	
*OAZ1* vs. *GUSB*	3.65	0.44		1.91	0.42	
*OAZ1* vs. *PPIA*	1.27	0.32		1.12	0.21	
*OAZ1* vs. *RPLP0*	4.61	0.34		3.24	0.36	
*OAZ1* vs. *TBP*	5.01	0.36		5.90	0.52	
*OAZ1* vs. *YWHAZ*	6.56	0.60		6.55	0.75	
*OAZ1* vs. *18S*	16.43	0.39	0.39	16.42	0.53	0.55
*GUSB* vs. *GAPDH*	5.89	0.38		4.73	1.33	
*GUSB* vs. *OAZ1*	3.65	0.44		1.91	0.42	
*GUSB* vs. *PPIA*	4.92	0.47		3.03	0.50	
*GUSB* vs. *RPLP0*	8.25	0.24		5.15	0.66	
*GUSB* vs. *TBP*	1.36	0.43		3.99	0.26	
*GUSB* vs. *YWHAZ*	2.91	0.61		4.64	0.42	
*GUSB* vs. *18S*	20.08	0.30	0.41	18.33	0.70	0.61
*TBP* vs. *GAPDH*	7.25	0.27		8.72	1.44	
*TBP* vs. *GUSB*	1.36	0.43		3.99	0.26	
*TBP* vs. *OAZ1*	5.01	0.36		5.90	0.52	
*TBP* vs. *PPIA*	6.28	0.26		7.03	0.64	
*TBP* vs. *RPLP0*	9.61	0.33		9.14	0.69	
*TBP* vs. *YWHAZ*	1.55	0.57		0.65	0.47	
*TBP* vs. *18S*	21.44	0.53	0.39	22.33	0.85	0.70
*YWHAZ* vs. *GAPDH*	8.80	0.52		9.37	1.48	
*YWHAZ* vs. *GUSB*	2.91	0.61		4.64	0.42	
*YWHAZ* vs. *OAZ1*	6.56	0.60		6.55	0.75	
*YWHAZ* vs. *PPIA*	7.83	0.55		7.68	0.75	
*YWHAZ* vs. *RPLP0*	11.16	0.45		9.79	0.98	
*YWHAZ* vs. *TBP*	1.55	0.57		0.65	0.47	
*YWHAZ* vs. *18S*	22.99	0.70	0.57	22.97	0.89	0.82

## References

[1] Jiang Y., Wang D., Blocki A., Tuan R.S., Lanza R., Langer R., Vacanti J.P., Atala A. (2020). Chapter 49—Mesenchymal stem cells in musculoskeletal tissue engineering. Principles of Tissue Engineering.

[2] Jaiswal N., Haynesworth S.E., Caplan A.I., Bruder S.P. (1997). Osteogenic differentiation of purified, culture-expanded human mesenchymal stem cells in vitro. J. Cell. Biochem..

[3] Mullis K., Faloona F., Scharf S., Saiki R., Horn G., Erlich H. (1986). Specific enzymatic amplification of DNA in vitro: The polymerase chain reaction. Cold Spring Harb. Symp. Quant. Biol..

[4] Mullis K.B., Faloona F.A. (1987). Specific synthesis of DNA in vitro via a polymerase-catalyzed chain reaction. Methods Enzym..

[5] Deepak S., Kottapalli K., Rakwal R., Oros G., Rangappa K., Iwahashi H., Masuo Y., Agrawal G. (2007). Real-Time PCR: Revolutionizing Detection and Expression Analysis of Genes. Curr. Genom..

[6] Higuchi R., Fockler C., Dollinger G., Watson R. (1993). Kinetic PCR analysis: Real-time monitoring of DNA amplification reactions. Biotechnology.

[7] Holland P.M., Abramson R.D., Watson R., Gelfand D.H. (1991). Detection of specific polymerase chain reaction product by utilizing the 5′-3′ exonuclease activity of Thermus aquaticus DNA polymerase. Proc. Natl. Acad. Sci. USA.

[8] Bustin S.A., Benes V., Garson J.A., Hellemans J., Huggett J., Kubista M., Mueller R., Nolan T., Pfaffl M.W., Shipley G.L. (2009). The MIQE guidelines: Minimum information for publication of quantitative real-time PCR experiments. Clin. Chem..

[9] Kozera B., Rapacz M. (2013). Reference genes in real-time PCR. J. Appl. Genet..

[10] Huggett J., Dheda K., Bustin S., Zumla A. (2005). Real-time RT-PCR normalisation; strategies and considerations. Genes Immun..

[11] Leong D.T., Gupta A., Bai H.F., Wan G., Yoong L.F., Too H.-P., Chew F.T., Hutmacher D.W. (2007). Absolute quantification of gene expression in biomaterials research using real-time PCR. Biomaterials.

[12] Wang L., Stegemann J.P. (2010). Extraction of high quality RNA from polysaccharide matrices using cetyltrimethylammonium bromide. Biomaterials.

[13] Yu C., Young S., Russo V., Amsden B.G., Flynn L.E. (2013). Techniques for the isolation of high-quality RNA from cells encapsulated in chitosan hydrogels. Tissue Eng. Part C Methods.

[14] Collins M.N., Birkinshaw C. (2013). Hyaluronic acid based scaffolds for tissue engineering—A review. Carbohydr. Polym..

[15] Behrendt P., Ladner Y., Stoddart M.J., Lippross S., Alini M., Eglin D., Armiento A.R. (2020). Articular Joint-Simulating Mechanical Load Activates Endogenous TGF-β in a Highly Cellularized Bioadhesive Hydrogel for Cartilage Repair. Am. J. Sports Med..

[16] Petta D., Armiento A.R., Grijpma D., Alini M., Eglin D., D’Este M. (2018). 3D bioprinting of a hyaluronan bioink through enzymatic-and visible light-crosslinking. Biofabrication.

[17] Vermeulen J., De Preter K., Lefever S., Nuytens J., De Vloed F., Derveaux S., Hellemans J., Speleman F., Vandesompele J. (2011). Measurable impact of RNA quality on gene expression results from quantitative PCR. Nucleic Acids Res..

[18] Ho-Pun-Cheung A., Bascoul-Mollevi C., Assenat E., Boissière-Michot F., Bibeau F., Cellier D., Ychou M., Lopez-Crapez E. (2009). Reverse transcription-quantitative polymerase chain reaction: Description of a RIN-based algorithm for accurate data normalization. BMC Mol. Biol..

[19] Schroeder A., Mueller O., Stocker S., Salowsky R., Leiber M., Gassmann M., Lightfoot S., Menzel W., Granzow M., Ragg T. (2006). The RIN: An RNA integrity number for assigning integrity values to RNA measurements. BMC Mol. Biol..

[20] Fleige S. (2006). RNA integrity and the effect on the real-time qRT-PCR performance. Mol. Asp. Med..

[21] Pérez-Novo C.A., Claeys C., Speleman F., Van Cauwenberge P., Bachert C., Vandesompele J. (2005). Impact of RNA quality on reference gene expression stability. Biotechniques.

[22] Vandesompele J., De Preter K., Pattyn F., Poppe B., Van Roy N., De Paepe A., Speleman F. (2002). Accurate normalization of real-time quantitative RT-PCR data by geometric averaging of multiple internal control genes. Genome Biol..

[23] Andersen C.L., Jensen J.L., Ørntoft T.F. (2004). Normalization of real-time quantitative reverse transcription-PCR data: A model-based variance estimation approach to identify genes suited for normalization, applied to bladder and colon cancer data sets. Cancer Res..

[24] Silver N., Best S., Jiang J., Thein S.L. (2006). Selection of housekeeping genes for gene expression studies in human reticulocytes using real-time PCR. BMC Mol. Biol..

[25] Cagnan I., Kaya F.A., Çetinkaya F.D., Özcan A. (2017). Stably expressed reference genes during differentiation of bone marrow-derived mesenchymal stromal cells. Turk. J. Biol..

[26] Ragni E., Viganò M., Rebulla P., Giordano R., Lazzari L. (2013). What is beyond a qRT-PCR study on mesenchymal stem cell differentiation properties: How to choose the most reliable housekeeping genes. J. Cell. Mol. Med..

[27] Jacobi A., Rauh J., Bernstein P., Liebers C., Zou X., Stiehler M. (2013). Comparative analysis of reference gene stability in human mesenchymal stromal cells during osteogenic differentiation. Biotechnol. Prog..

[28] He T., Huang Y., Chak J.C., Klar R.M. (2018). Recommendations for improving accuracy of gene expression data in bone and cartilage tissue engineering. Sci. Rep..

[29] Barber R., Harmer D.W., Coleman R.A., Clark B.J. (2005). GAPDH as a housekeeping gene: Analysis of GAPDH mRNA expression in a panel of *72* human tissues. Physiol. Genom..

[30] Rauh J., Jacobi A., Stiehler M. (2015). Identification of stable reference genes for gene expression analysis of three-dimensional cultivated human bone marrow-derived mesenchymal stromal cells for bone tissue engineering. Tissue Eng. Part C Methods.

[31] Quiroz F.G., Posada O.M., Gallego-Perez D., Higuita-Castro N., Sarassa C., Hansford D.J., Agudelo-Florez P., López L.E. (2010). Housekeeping gene stability influences the quantification of osteogenic markers during stem cell differentiation to the osteogenic lineage. Cytotechnology.

[32] Chooi W.H., Zhou R., Yeo S.S., Zhang F., Wang D.-A. (2013). Determination and validation of reference gene stability for qPCR analysis in polysaccharide hydrogel-based 3D chondrocytes and mesenchymal stem cell cultural models. Mol. Biotechnol..

[33] de Jonge H.J., Fehrmann R.S., de Bont E.S., Hofstra R.M., Gerbens F., Kamps W.A., de Vries E.G., van der Zee A.G., te Meerman G.J., ter Elst A. (2007). Evidence based selection of housekeeping genes. PLoS ONE.

[34] Gubern C., Hurtado O., Rodríguez R., Morales J.R., Romera V.G., Moro M.A., Lizasoain I., Serena J., Mallolas J. (2009). Validation of housekeeping genes for quantitative real-time PCR in in-vivo and in-vitro models of cerebral ischaemia. BMC Mol. Biol..

[35] Halouani A., Jmii H., Michaux H., Renard C., Martens H., Pirottin D., Mastouri M., Aouni M., Geenen V., Jaïdane H. (2020). Housekeeping Gene Expression in the Fetal and Neonatal Murine Thymus Following Coxsackievirus B4 Infection. Genes.

[36] Brinkhof B., Jia H., Zhang B., Cui Z., Ye H., Wang H. (2018). Improving characterisation of human Multipotent Stromal Cells cultured in 2D and 3D: Design and evaluation of primer sets for accurate gene expression normalisation. PLoS ONE.

[37] Jeon R.-H., Lee W.-J., Son Y.-B., Bharti D., Shivakumar S.B., Lee S.-L., Rho G.-J. (2019). PPIA, HPRT1, and YWHAZ Genes Are Suitable for Normalization of mRNA Expression in Long-Term Expanded Human Mesenchymal Stem Cells. Biomed Res. Int..

[38] Cassinelli C., Morra M., Pavesio A., Renier D. (2000). Evaluation of interfacial properties of hyaluronan coated poly (methylmethacrylate) intraocular lenses. J. Biomater. Sci. Polym. Ed..

[39] Matsumoto K., Li Y., Jakuba C., Sugiyama Y., Sayo T., Okuno M., Dealy C.N., Toole B.P., Takeda J., Yamaguchi Y. (2009). Conditional inactivation of Has2 reveals a crucial role for hyaluronan in skeletal growth, patterning, chondrocyte maturation and joint formation in the developing limb. Development.

[40] Sathyendra V., Darowish M. (2013). Basic Science of Bone Healing. Hand Clin..

[41] Cheng A., Genever P.G. (2010). SOX9 determines RUNX2 transactivity by directing intracellular degradation. J. Bone Miner. Res..

[42] Zhou G., Zheng Q., Engin F., Munivez E., Chen Y., Sebald E., Krakow D., Lee B. (2006). Dominance of SOX9 function over RUNX2 during skeletogenesis. Proc. Natl. Acad. Sci. USA.

[43] Zou L., Zou X., Chen L., Li H., Mygind T., Kassem M., Bünger C. (2008). Effect of hyaluronan on osteogenic differentiation of porcine bone marrow stromal cells in vitro. J. Orthop. Res..

[44] Dy P., Wang W., Bhattaram P., Wang Q., Wang L., Ballock R.T., Lefebvre V. (2012). Sox9 directs hypertrophic maturation and blocks osteoblast differentiation of growth plate chondrocytes. Dev. Cell.

[45] Monaco G., El Haj A.J., Alini M., Stoddart M.J. (2020). Sodium Hyaluronate Supplemented Culture Media as a New hMSC Chondrogenic Differentiation Media-Model for in vitro/ex vivo Screening of Potential Cartilage Repair Therapies. Front. Bioeng. Biotechnol..

[46] Burdick J.A., Prestwich G.D. (2011). Hyaluronic acid hydrogels for biomedical applications. Adv. Mater..

[47] Armiento A.R., Hatt L.P., Sanchez Rosenberg G., Thompson K., Stoddart M.J. (2020). Functional Biomaterials for Bone Regeneration: A Lesson in Complex Biology. Adv. Funct. Mater..

[48] Burdick J.A., Chung C., Jia X., Randolph M.A., Langer R. (2005). Controlled degradation and mechanical behavior of photopolymerized hyaluronic acid networks. Biomacromolecules.

[49] Smeds K.A., Pfister-Serres A., Miki D., Dastgheib K., Inoue M., Hatchell D.L., Grinstaff M.W. (2001). Photocrosslinkable polysaccharides for in situ hydrogel formation. J. Biomed Mater. Res..

[50] Gardner O.F., Alini M., Stoddart M.J. (2015). Mesenchymal Stem Cells Derived from Human Bone Marrow. Methods Mol. Biol..

[51] Phelipe Hatt L., Thompson K., Muller W.E.G., Stoddart M.J., Armiento A.R. (2019). Calcium Polyphosphate Nanoparticles Act as an Effective Inorganic Phosphate Source during Osteogenic Differentiation of Human Mesenchymal Stem Cells. Int. J. Mol. Sci..

[52] Schildberg T., Rauh J., Bretschneider H., Stiehler M. (2013). Identification of suitable reference genes in bone marrow stromal cells from osteoarthritic donors. Stem Cell Res..

